# Digital Mapping of Central Asian Foods: Towards a Standardized Visual Atlas for Nutritional Research

**DOI:** 10.3390/nu17213315

**Published:** 2025-10-22

**Authors:** Zhuldyz Omarova, Bibinur Nurmanova, Aibota Sanatbyek, Huseyin Atakan Varol, Mei-Yen Chan

**Affiliations:** 1 School of Medicine, Nazarbayev University, Astana 010000, Kazakhstan; 2 Institute of Smart Systems and Artificial Intelligence, Nazarbayev University, Astana 010000, Kazakhstanahvarol@nu.edu.kz (H.A.V.)

**Keywords:** portion size estimation, dietary assessment, Central Asia, visual food atlas, culinary heritage

## Abstract

**Background/Objectives:** Portion size estimation is important for dietary assessment and nutrition research, but has remained understudied in Central Asia, a region characterized by red meat-rich diets and high rates of diet-related noncommunicable diseases (NCDs). Therefore, this study aimed to develop a digital visual food atlas for Central Asian cuisine that would provide high-quality images of commonly consumed foods and beverages, while special focus was given to meat dishes that were not present in previous atlases. **Methods**: Foods were selected based on the Central Asian Food Dataset (CAFD) and Central Asian Food Scenes Dataset (CAFSD) and photographed in three portion sizes: small, average, and large. There were nine broad categories: main dishes, soups, meat dishes, salads, snacks, side dishes, bakery and bread, desserts, and beverages. Similar settings were preserved for each photograph: the 60° angle, sufficient lighting, and food setup (including reference objects like utensils, a ruler, and a neatly folded napkin). **Results**: The final digital visual food atlas comprised 115 items (95 food series, 20 beverage guides), with 12 meat-based dishes, reflecting the central role of meat in regional diets. Each entry included portion weights and names in both English and local languages, improving cultural and linguistic relevance. The digital format with clear labeling ensured accessibility on web and mobile platforms. **Conclusions**: This was the first digital visual food atlas developed for Central Asia, providing standardized portion-size references. The atlas offered a practical tool for dietary assessment, with applications in nutrition research, mobile health technologies, artificial intelligence (AI)-driven portion estimation, and policy development.

## 1. Introduction

Portion size estimation is an important part of dietary assessment and nutrition research, which provides insights into the relationship between diet and health outcomes [1]. The accuracy of portion size estimation determines the subsequent nutrient intake calculations [2,3]. Inaccuracies can cause considerable errors in food intake data and distort the interpretation of the results [1]. 

To address these challenges, portion size estimation aids (PSEAs), including hand-based estimations, cups and spoons, food scales, digital apps, and visual portion guides, have been used as supplementary tools to improve accuracy. Among these, portion size estimation elements (PSEEs) based on food images have shown better accuracy compared to traditional tools such as physical food models or cups and spoons [1]. For instance, food atlases, structured collections of food photographs, are commonly used in large-scale dietary surveys [4,5,6,7]. These atlases typically include reference objects and capture food from multiple angles to support visual perception and portion estimation [5,8]. Food atlases often display multiple portion options (usually three to five images per food item), enabling users to make more accurate comparisons [3,7]. 

Despite their usefulness, food atlases are subject to variability due to cultural diversity in dietary practices, food preparation, and serving styles [2]. Moreover, geographical locations and climatic conditions of a region significantly impact the food consumed in a particular area. To address this, region-specific food atlases have been developed globally. For example, European countries such as Greece [6], Italy [9], and the United Kingdom (UK) [10] have developed regional atlases that include commonly consumed foods and depict them in various portions. Another atlas was designed for the Balkan region, combining the dietary patterns of Serbia, Montenegro, and Bosnia and Herzegovina [5]. Similarly, national food atlases were developed in Asia and the Middle East, in countries such as China, India, and the United Arab Emirates (UAE), being tailored to their cuisines [4,7,11]. In Africa, emerging food atlases have been developed in countries like Kenya and Tunisia, while there are fewer examples in the Americas and Oceania [4]. These digital tools aid in preserving culinary heritage, which encompasses region-specific food traditions and everyday practices surrounding food. Such digitization of cultural experiences has enabled wider participation, access, creation, production, and sharing of knowledge, while strengthening cultural identity. One such example is the Mediterranean Diet, which is recognized as an Intangible Cultural Heritage of Humanity by UNESCO [12]. In addition to its cultural and nutritional importance, it has great potential in addressing significant global challenges, such as hunger, sustainable agriculture, food safety, biodiversity conservation, and food waste management [12,13].

Cultural heritage is an umbrella term that includes traditions, rituals, crafts, languages, and social practices that shape community identity [12]. Food and dietary practices, as part of culinary heritage, embody cultural values through preparation methods, communal dining practices, and symbolic meanings [12]. Documenting foods and customary practices is therefore not only an important part of nutrition surveillance but also an integral part of preserving culinary heritage.

Central Asia currently lacks a dedicated food atlas despite its distinctive and unique dietary patterns, characterized by high consumption of red meat (lamb, beef, and horse) and limited seafood [14,15]. This is of particular concern given the region’s high burden of diet-related noncommunicable diseases (NCDs), including cardiovascular disease, type 2 diabetes, and obesity, as well as some cancers [16,17,18]. Without such a dedicated atlas, accurate or tailored dietary assessment, pivotal to addressing personalized nutrition, will be challenging to conduct. Dietary risks, such as high consumption of calorie-dense and sugary foods and red and processed meat, are associated with higher risks of developing NCD and different types of cancers [19,20]. Health risks associated with red and processed meat consumption arise from their high levels of cholesterol and saturated fats, as well as harmful compounds added or formed during processing and cooking, such as *N*-nitroso compounds and polycyclic aromatic hydrocarbons, and oxidation products generated during metabolism [20]. Although red meat and processed meat products cannot be totally eliminated from the diets of the populations, it is necessary to study this aspect of nutrition intake more critically in this region. Despite the knowledge about the role of diet in the prevention of NCDs, Central Asia has difficulties in monitoring and evaluating population-level diet behavior. The essential barrier is the absence of standardized instruments and methods to conduct large-scale dietary surveys. In addition, systematic surveillance systems that have studied dietary trends, food consumption patterns, and other affiliated health outcomes are currently underdeveloped compared to other countries and the existing US National Health and Nutrition Examination Survey (NHANES) [21] and the European Food Safety Authority European Union Menu (EFSA EU-Menu) Program [22]. This gap underscores the need to address the increased burden of diet-related diseases in Central Asia, beginning with the development of dietary surveillance methods based on validated assessment tools.

In this regard, digital tools such as food image recognition and artificial intelligence (AI)-enabled applications, as well as smartphone devices, offer promising solutions to monitor dietary intakes, enhance the accuracy of dietary assessments, and provide personalized recommendations, reducing reliance on memory and improving user compliance [5]. Previous studies have shown that food atlases contribute to nutrition research by serving as foundational tools for developing automated dietary assessment systems and national nutrition policies [4]. Food atlases are widely used in nutritional surveys, significantly improving the accuracy of 24-h recalls (24HR), food frequency questionnaires (FFQ), and nutrition surveys [4]. In clinical settings, food atlases help dietitians and healthcare providers assess and counsel patients more effectively, supporting better nutritional guidance and care [4]. 

To the best of the authors’ knowledge, one of the novel aspects of this piece of research was that there are not any records of these in which typical meal portion sizes, the types and volumes of serving ware, accurate weight and volume measurements, multilingual food names rooted in local languages, and the context of cultural norms of communal dining are included. Beyond their practical role in dietary research, these elements are also needed to represent culinary heritage, including how food is served, shared, and named in everyday life, which forms an integral part of culinary heritage.

To address this gap, this study aimed to develop a digital visual food atlas for Central Asian cuisine. The atlas presented high-quality images of commonly consumed foods, organized by portion sizes, with each portion clearly labeled with its corresponding weight and volume. It also included meat dishes that were underrepresented in previous atlases. To the best of the authors’ knowledge, this atlas was the first in the region to present a standardized reference tool for more accurate dietary assessments, contributing to better insights into diet and nutrition in Central Asia, while supporting the preservation of the region’s culinary heritage.

## 2. Materials and Methods

### 2.1. Data Source and Food Selection

The foundation of the digital visual food atlas was based on two complementary datasets: the Central Asian Food Dataset (CAFD) [23] and the Central Asian Food Scenes Dataset (CAFSD) [24]. Both were developed by Nazarbayev University and are housed at the Institute of Smart Systems and Artificial Intelligence (ISSAI).

The CAFD provides over 16,000 web-scraped images of 42 distinct food categories, representing the culinary diversity of Central Asia, especially traditional dishes and commonly consumed foods from Kazakhstan, Uzbekistan, Kyrgyzstan, Turkmenistan, and Tajikistan. It is openly available on GitHub (https://github.com/IS2AI/Central-Asian-Food-Dataset, commit 430c438, accessed on 15 April 2025) [23].

The CAFSD expanded this coverage and contains 21,306 images with 69,856 instances across 239 food classes, organized into 18 broad categories benchmarked against the FAO/WHO Global Individual Food Tool. The dataset is available on HuggingFace (https://huggingface.co/datasets/issai/Central_Asian_Food_Scenes_Dataset, commit adcf1182beb2b5e85d492f1e22d1f655b18e49bf, accessed on 15 April 2025). Images were sourced from three streams: 15,939 web-scraped images (Google, YouTube, and Yandex), 2324 everyday-life photographs, and 3043 video frames [24].

For the purposes of the atlas, food items were selected from both datasets to ensure cultural representativeness, with emphasis on commonly consumed dishes and beverages in Kazakhstan, Uzbekistan, Kyrgyzstan, Turkmenistan, and Tajikistan. Although the CAFD and CAFSD are no longer being actively updated, they provide a comprehensive collection of traditional and commonly consumed foods from the region. The datasets include images of both popular and underrepresented foods, such as plov (pilaf), manty (dumplings), and shashlik (grilled meat), as well as more region-specific dishes like asip (traditional meat sausage). This diversity in food types and preparation methods makes the datasets essential for developing a digital visual food atlas that accurately reflects Central Asian cuisine [23,24]. By focusing on foods uniquely tied to Central Asian cuisine, we selected the commonly consumed food items usually served as meals, excluding ready-to-eat products and items typically consumed without any preparation. 

It should be noted that CAFD and CAFSD are not designed for lay public use in dietary assessment, as they do not contain standardized portion sizes. These datasets contain web-scraped food images that were used to train models for food object detection [23,24]. All photographs with three portion sizes included in this Central Asian Digital Visual Food Atlas were newly produced by the team under standardized conditions.

### 2.2. Food Categorization

The CAFD includes 42 food classes, while the CAFSD contains 239 food classes organized into 18 broad categories [23,24]. These structures provided detailed labeling of foods but were not directly applied in the atlas. Instead, for the purposes of the study, a simplified categorization system was developed to construct the atlas. Food items were grouped into broad categories such as main dishes, side dishes, soups, salads, and meat dishes, with differentiation made between liquid and solid foods. This classification was based on the kind of dish, preparation techniques, and the respective role it performs in meals. Other categories included snacks and bakery products, as well as beverages, while desserts were added as categories to represent sweets often served after main meals. This approach ensured that the categorization reflected cultural eating contexts and serving practices, while also facilitating portion size estimation in the digital visual food atlas.

### 2.3. Determination of the Type of Photographs

Based on previous research indicating that series-based images provide more accurate portion size estimations than single images [25], the digital visual food atlas was designed to present each food item in a sequence of three photographs rather than in isolated representations. These photographs corresponded to small, average, and large portions, enabling more precise visual comparison of portion size variations. This method ensured that users could better estimate the range of portion sizes typically encountered in everyday meals. In contrast, beverages were presented with a single guide image, representing one size, as their portions are more standardized and less variable compared to food items.

### 2.4. Display of Items

**Figure 1:**
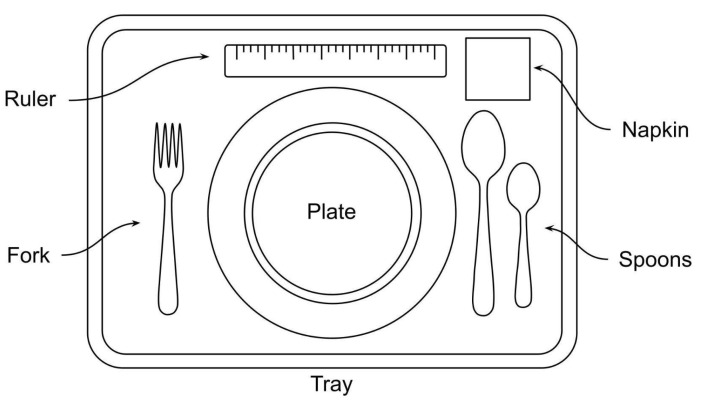
Schematic tray setup with plate, utensils, and reference objects.

To improve perception of portion size, a visual setup was designed, presenting common dining items relative to familiar household objects such as plates, utensils, and cups. In the center of the setup, a rectangular tray held a round plate, providing a clear frame for the food items and reinforcing their relative sizes. The fork and spoons were placed symmetrically on either side of the plate, while a ruler was positioned above the plate as the reference scale, providing a practical visual measure for the depiction of portion sizes. To further contextualize the display, a neatly folded napkin was placed in the upper-right corner of the tray. Minor variations in the placement of utensils, napkins, or other items occurred depending on the specific food arrangement, but these did not affect the clarity or purpose of the visual reference. The schematic tray setup is shown in Figure 1. 

### 2.5. Determination of Portion Sizes

Each food item included in the digital visual food atlas was depicted using three standard portion sizes: small, average, and large. These portion sizes were determined through a comprehensive approach, which combined commonly sold portion sizes, standard portioning principles from previous dietary assessment studies, and portion size coefficients to align with real-world consumption patterns. In this model, the average portion was designated as the baseline (1.0), the large portion was scaled up by a coefficient of 1.5, and the small portion was adjusted to 0.5. This method followed established portion modeling techniques, such as those described by previous researchers [5]. The determination of large portions was based on portion sizes commonly served in public eating venues, food services, and culturally relevant meal settings. Portion sizes were not directly linked to the CAFD or CAFSD images.

### 2.6. Preparation of Tableware

**Figure 2:**
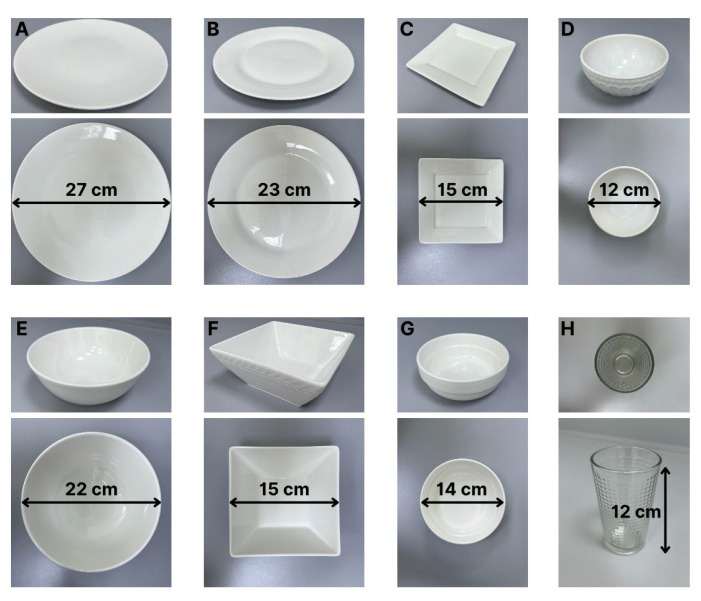
Examples of tableware: (**A**) 27 cm diameter round plate; (**B**) 23 cm diameter round plate; (**C**) 15 × 15 cm square plate; (**D**) tea bowl; (**E**) large soup bowl; (**F**) salad bowl; (**G**) small soup bowl; and (**H**) 250 mL glass.

The tableware for the digital visual food atlas was selected to accurately reflect a variety of commonly used dining items in Central Asian households. This selection included a variety of plates, bowls, and glasses that represent typical household tableware, ensuring a broad representation of the different types of plates and containers used to serve meals (see Figure 2). This variety ensured that the food atlas could be used to estimate portion sizes across a wide range of real-world meal presentations. Table 1 summarizes the measurement of eight household items.

### 2.7. Weighing of Food Samples

Before photography, all food portions were pre-weighed using a digital scale (Vitek VT-8020, manufactured by VITEK/Golder Electronics, Moscow, Russia; produced in China; sourced from Technodom, Almaty, Kazakhstan), with a precision of ±0.1 g. This careful measurement process was essential to accurately represent the portion sizes in the food atlas, ensuring that the visual representations aligned with the actual weight of the portions.

### 2.8. Photography and Image Processing

**Figure 3:**
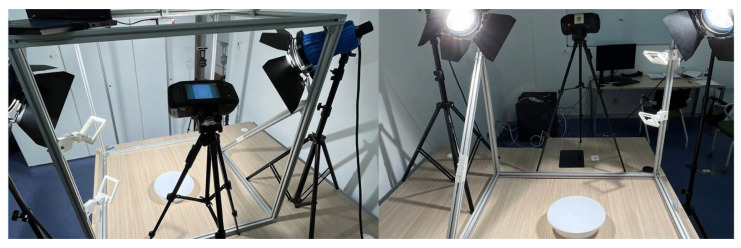
Camera setup for image collection.

Food photographs were acquired with the RGB (Red, Green, Blue) camera of a smartphone (Apple iPhone 13, manufactured by Apple Inc., Cupertino, CA, USA; assembled in China; 3024 × 4032 resolution, JPEG format). The photos were taken at the same angle (60°) and in the same lighting and food setup to ensure consistency, as shown in Figure 3. Two softboxes, 58 watts each, were used to ensure uniform lighting conditions and prevent shadows. Previous studies used the 45° angle to reflect the width and depth of the food items depicted specifically for each portion size, while we adjusted this angle to achieve a higher representation, considering the distance between the camera and the food setup [26,27].

### 2.9. Digital Visual Food Atlas Development

The Digital Visual Food Atlas was created using Canva (version 2.310.0), where standardized food images, descriptive texts, and portion weight labels were systematically arranged into a clear, structured layout. Each food item was depicted in three portions, with consistent formatting and vertical alignment to facilitate easy comparison.

## 3. Results

The completed digital visual food atlas included 115 distinct foods and beverages commonly consumed across Central Asia, encompassing Kazakhstan, Uzbekistan, Kyrgyzstan, Turkmenistan, and Tajikistan. Of 115 items included in the food atlas, 95 were photographed as a series of photographs of foods and 20 as guide photographs of beverages. Food names were either translated into English when a clear, relevant equivalent existed or transcribed into the Latin alphabet when translation would lose cultural nuance or no precise equivalent existed. This allowed addressing the region’s linguistic diversity.

The collection featured a mix of traditional dishes (e.g., plov/pilaf, beshbarmak, and lagman), everyday foods, and internationally popular items (e.g., pizza and burgers). Foods were categorized into nine sections—Main Dishes, Soups, Meat Dishes, Salads, Snacks, Bakery and Bread, Side Dishes, Beverages, and Desserts—facilitating easy navigation. Each category contained multiple representative foods, such as approximately twelve soups (ranging from borscht to shorpa), nearly twenty main courses, and a diverse selection of breads, drinks, and sweets. Approximately 30 items included meat as a side ingredient or the main component, reflecting the significance of meat-based foods within the regional diets. This classification enabled users to quickly locate foods of interest and compare items within the same category. A complete list of the food items included, along with their descriptions and names in local languages, where Kaz, Uzb, Kir, Tgk, and Tuk represent Kazakh, Uzbek, Kyrgyz, Tajik, and Turkmen, respectively, is provided in Table 2.

**Figure 4:**
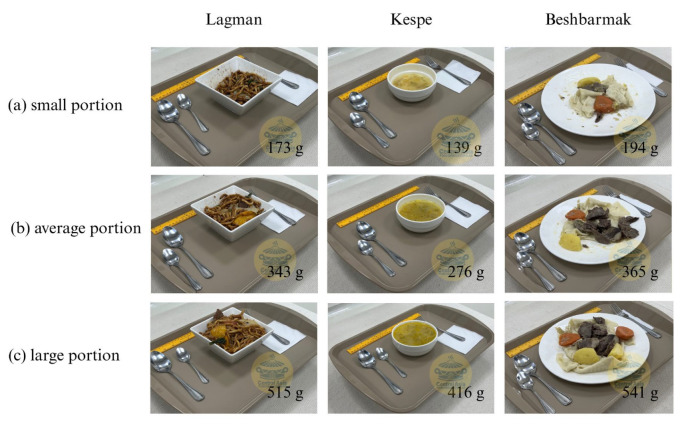
Examples of food photo series taken for the visual atlas in: (**a**) small; (**b**) average; and (**c**) large portions.

Figure 4 illustrates examples of food photo series used in the digital visual food atlas, depicting three portion sizes: (a) small portion, (b) average portion, and (c) large portion. Each image shows the food served on a standard tray with accompanying utensils and a reference ruler for scale. The series captures variations in portion sizes, providing clear visual comparisons to aid in accurate portion size estimation.

**Figure 5:**
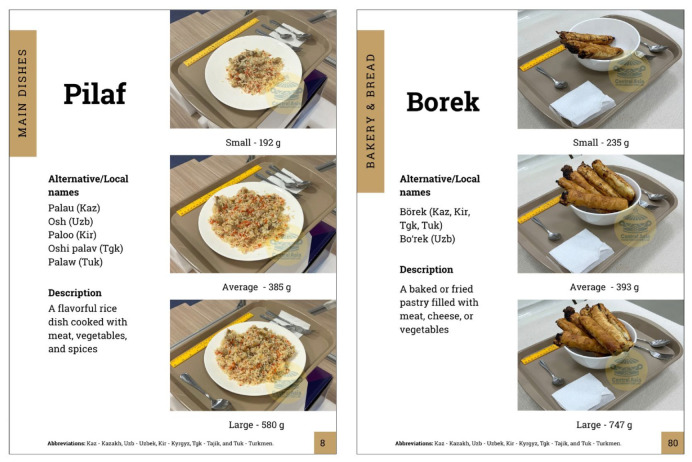
Example page of the digital visual food atlas.

Each food item in the atlas was depicted with high-quality photographs illustrating three portion sizes: small, average, and large. For example, as shown in Figure 5, a plate such as pilaf was presented on a standard plate in three quantities, with the exact weight of each portion marked (e.g., 192 g, 385 g, and 580 g) to serve as a reference. This approach provided users with a visual range of portion sizes, allowing them to compare their intake with the closest image for an accurate estimation. All images were produced using standardized methods, ensuring consistency across the atlas: foods were photographed from similar angles, on neutral backgrounds, and typically placed on local dinnerware with familiar utensils or measurement references for scale.

The atlas was organized in a user-friendly format, with a content page that lists all foods by category. Each food entry included portion images, the food’s name (in both local and English names, as applicable), and a brief descriptive text, which helps facilitate comparison and recognition between different foods. The digital design ensured accessibility on web or mobile platforms, with an intuitive interface that allows users, including researchers, dietitians, and the general public, to browse categories, search for specific foods, and zoom in on images for greater details.

The interface prioritized clarity through simple labels, high-contrast images, and logical grouping, making it suitable for users of various ages and educational backgrounds. The atlas is available in English and is optimized for use on standard smartphones. The digital format also allows for easy updates and expansions over time, reducing barriers to access compared to a paper-based format, which is restricted to specific locations. In general, the key features of the atlas were its comprehensive coverage of Central Asian foods, standardized portion-size images, clear organization, and a design focused on engaging and convenient use by a broad audience.

## 4. Discussion

To our knowledge, this is the first comprehensive digital visual food atlas to present commonly consumed food products from Central Asian cuisine. Although some food atlases had been published for dietary assessments in various regions, few included meat products. The food list included in our atlas was based on the CAFD and CAFSD to ensure both cultural relevance and accuracy.

While many food atlases relied on population-based weighted dietary records (DRs) to determine food types and portion sizes, the existing datasets, like CAFD and CAFSD, were considered a reliable representation of the region’s typical foods [23,24]. This method eliminated the necessity for particular dietary recalls or surveys by using an existing, properly selected dataset to reflect local food habits with daily and seasonal variations. 

The completed digital visual food atlas included 115 distinct items commonly consumed across Central Asia, including 95 photo series of food, showing small, average, and large portions, and 20 guide photographs (photos of beverages). There are no current established gold standards as to what foods or how many portions should be in a visual food atlas. For instance, the Tunisian food atlas [27] included digital photographs of three portion sizes and 400 food items, while the food atlas for the Balkan region encompassed four portion sizes and 135 food items [5]. In the UK, the food compendium was designed for children’s and adolescents’ diets, combining the most commonly consumed 104 foods and depicting them in seven portions [10]. Similarly, the food photography manuals developed in Sri Lanka, the UAE, Malaysia, and other countries provided varying portion sizes and numbers of food items [11,28,29]. According to previous findings, people tend to make errors in estimating many food groups, including foods without regular shape or consistency, liquids, and spreads [27,30,31]. Additionally, the most common issue is underestimating the amount of food consumed. Therefore, visual food atlases have been developed to aid visual perception and direct people’s judgment in dietary assessments, reducing variability and extreme errors. The serving size depictions in the atlas were not validated with consumers as part of this study. However, this is a critical next step, and we plan to address it in our subsequent work. A follow-up publication will focus on consumer validation to assess the accuracy and applicability of the atlas in dietary intake assessment.

The strength of the Central Asian visual food atlas is that this resource can be applied in various contexts to improve dietary assessment, technology integration, and education. This version was published in a simplified, accessible format to make it usable by a broader group of target users in different settings. Other potential applications of this tool include:

**Dietary assessment in research and public health.** The digital visual food atlas can be used by nutritionists and epidemiologists in combination with dietary assessment tools, including 24HRs, FFQs, and other surveys, to enhance portion estimations in Central Asian populations. For instance, respondents can select an image that matches the portion they consumed without relying on memory or verbal descriptions. Previous findings demonstrated the increased correlation between portion images and actual consumed amounts when using a food atlas [7]. Considering that a culturally tailored atlas can contribute to more reliable data on food intake, these results might be further used to examine diet-related health conditions in Central Asian populations. Moreover, public health agencies could use the atlas in large-scale nutrition surveys. These data will allow monitoring of meat consumption and NCDs epidemiology to obtain more accurate health data and examine their relationship, thereby informing targeted interventions.

**Integration with nutrition apps and AI portion estimation**. The digital visual food atlas can be integrated into mobile diet-tracking apps or clinical nutrition software as a built-in portion guide, enabling semi-automated dietary logging. Consumers could rely on the atlas images when recording their diet by matching portion sizes and making them less error-prone. Additionally, the image database (with grounded weights) can facilitate the performance of machine learning models by teaching a machine vision algorithm to distinguish food types and estimate their portions. For instance, a library of ~200 photos of food was included in the online diet survey developed in Australia to estimate portion sizes [32]. Similarly, in Central Asia, the integration of this atlas into mobile apps or other technological tools could improve dietary control and personalize further guidelines. For instance, the user could be informed whether a selected portion is smaller or larger than recommended or if the daily or weekly meat consumption is acceptable. This combination of visual aids with technology might create a modern, systematic surveillance system that could study dietary trends, food consumption patterns, and other affiliated health outcomes.

**Nutrition education**. This developed tool might also be used for educational purposes to guide professionals. Health specialists and dietitians could use the images during counseling or workshops to demonstrate the difference between small and large portions of local foods (for example, showing the difference between serving sizes of plov). Such visuals might enhance portion size perception and teach individuals to monitor their intake.

**Practical Implications for Researchers, Governments, and Non-Govermental Organizations (NGOs).** The Central Asian Digital Visual Food Atlas provides a culturally specific tool that can be applied across multiple sectors. For researchers, it provides a standardized reference for dietary assessment in both epidemiological and clinical studies, reducing errors in portion size estimation and improving data quality. The atlas can help governments design and evaluate food-based dietary guidelines that are consistent with local dietary habits, inform national surveillance of diet and noncommunicable diseases, and develop culture-specific dietary recommendations and policies. For NGOs engaged in food security, nutrition education, and health promotion, the atlas represents an accessible resource to facilitate community-based interventions, training, and awareness campaigns. Its digital format enhances scalability, allowing integration into mobile technologies and AI-based dietary applications, thereby extending its practical use beyond academic research into public health practice and grassroots initiatives.

Despite its usefulness, the food atlas project faced several challenges and limitations that need to be acknowledged and addressed for future work:

**Cultural food diversity and regional variation.** Central Asian cuisine includes diverse items. Their recipes, ingredients, and typical serving sizes might vary by location or even household. Therefore, some lesser-known regional foods might be omitted, while the tableware was carefully selected to align with culturally appropriate norms.

**Technical limitations in image standardization.** Designing a standardized set of photographs of more than a hundred foods was a technical challenge. Minor differences in presentation (plate shape, camera height, lighting, angle, and background) could affect the portion’s appearance. Also, in some images, parts of the food items or accompanying utensils might have been partially hidden due to occlusion. To address these, a camera setup (e.g., angle/distance, a reference ruler in shots, consistent lighting) was fixed, but the shape and food consistency were not the same, as some spread out on the plate, while others stacked high. Therefore, the digital visual food atlas should be regularly updated to include new foods or recipe variations.

**Food variety and processing differences.** The differences in ingredients, cooking, and serving could affect the estimation and visual presentation of portions. An example would be that soups might come in different densities, and other individual items may not be seen under the broth. Similarly, standard composite dishes in the digital visual food atlas might differ from one person’s serving (e.g., more meat vs. potato), affecting the final nutritional value. The control of meat-based products also might have been problematic because meat dishes might contain bones or fat not counted in the edible portion weight. Also, certain foods, such as cereal grains and meat, change weight or volume upon processing. Some atlases addressed this by showing both raw and cooked weights [33].

Therefore, although the digital visual food atlas provides a solid reference, it cannot capture every change in food variety or individual dietary habits. The current version provides visual representations and names of foods but does not contain information on their nutritional composition since this research work is in progress. As a result, the atlas cannot directly be used for nutrient intake estimation. These limitations should be considered in future applications and possibly adjusted to the estimations to achieve better accuracy.

These limitations do not diminish the importance of the creation of a culturally adjusted digital visual food atlas of Central Asia. In the absence of such a reference, local dietary evaluations would have involved generic portion sizes of other cuisines, and this would have introduced more estimation bias and the limitation of lower cultural applicability. Although there may be a variation in the recipes or presentation, the atlas offered a visual standard point to assist the reporting based on memory and enhance the estimation of portion sizes. Moreover, the digital form can be updated periodically to accommodate new foods, changing food consumption patterns, and the incorporation of technology.

## 5. Conclusions

In this study, we created a digital visual food atlas with standardized images of Central Asian food, including the most widely consumed foods and beverages. The atlas can be used in various contexts: to improve dietary assessment through research and public health studies, inclusion in mobile nutrition apps and AI-powered portion estimating systems, nutrition education, and culturally appropriate policy work. Along with technology, it could also contribute to the systematic surveillance of the diet trend, food consumption patterns, and the related health outcomes, like the patterns of meat consumption in the region and NCD epidemiology, by integrating visual support. Despite the cultural and technical variability, the atlas provides much-needed cross-referenced visual information about the region and can be continuously updated for improved accuracy.

## Figures and Tables

**Table 1:** List of tableware.

Tableware	Top Width (cm)	Bottom Width (cm)	Height (cm)
Large Soup Bowl	22.0	15.5	8.2
Salad Bowl	15.0	8.5	6.0
Tea Bowl	12.0	5.5	6.0
Small Soup Bowl	14.0	9.5	5.5
Square Plate	15.0	-	1.5
Round Plate	23.0	-	2.0
Big Round Plate	27.0	-	3.0
Glass	7.0	5.5	12.0

**Table 2:** List of included food items in the digital visual food atlas, their alternative/local names, and descriptions.

Category	Dish Name	Alternative/Local Name	Description
Main Dishes	Pilaf	Palau (Kaz), Osh (Uzb), Paloo (Kir), Oshi palav (Tgk), Palaw (Tuk)	A flavorful rice dish cooked with meat, vegetables, and spices
	Dumplings	Tushpara (Kaz), Chuchvara (Uzb), Chuchpara (Kir), Tushbera (Tgk), Borek (Tuk)	A dish made of dough with a meat filling in the form of small parcels, boiled and served with broth. Dumplings can also be steamed, boiled, or fried
	Pizza	-	A dish consisting of a baked flatbread topped with tomato sauce, cheese, and various toppings
	Burger	-	A sandwich with a cooked patty (often beef), served with lettuce, tomato, and sauces inside a bun
	English Breakfast	Ağylşyn tañğı asy (Kaz), Ingliz nonushtasi (Uzb), Anglís ertenge menenki tamagy (Kir), Subhona anglisi (Tgk), Iňlis ertirlik (Tuk)	A hearty meal including eggs, bacon, sausage, baked beans, toast, and often mushrooms and tomatoes
	Cabbage Rolls	Russian golubtsi (Kaz, Uzb, Kir, Tgk, Tuk)	Cooked cabbage leaves stuffed with a mixture of meat and rice, then simmered in a tomato-based sauce
	Fried Lagman	Qyyrylgan lagman (Kaz), Qovurilgan lagman (Uzb), Lagmoni biryon (Tgk), Gowrulan lagman (Tuk), Kuurulgan laghman (Kir)	Stir-fried noodles with meat and vegetables
	Quesadilla	Kesadilla (Kaz, Kir, Uzb), Kesadiliya (Tgk), Kwesadilla (Tuk)	Warm corn or wheat flour tortillas filled with various ingredients, folded in half, and pan-fried
	Fettuccini	-	Long, flat Italian pasta, often served with creamy sauces like Alfredo
	Rice Porridge	Kurish botqasy (Kaz), Guruch bo‘tqasi (Uzb), Kürüch botkosu (Kir), Shir-birinch (Tgk), Tüwi botgasy (Tuk)	A thick porridge cooked with milk (or water) and rice grains, slowly simmered over low heat
	Lagman	Lagman (Kaz, Uzb, Tgk, Tuk), Laghman (Kir)	A dish made with meat, vegetables, and hand-pulled long noodles
	Fried Dumplings	Qyyrylgan tushpara (Kaz), Qovurilgan chuchvara (Uzb), Kuurulgan chuchpara (Kir), Tushbera biryon (Tgk), Gowrulan borek (Tuk)	Made from dough filled with meat, served fried with a sauce
	Rice with Meatballs	Küris frikadelkamen (Kaz), Guruch go‘sht sharchalari bilan (Uzb), Kürüch et shary menen (Kir), Birinj bo kufta (Tgk), Tüwi et köftesi bilen (Tuk)	Minced meat or fish formed into balls and served with rice as a side dish
	Beshbarmak	Beshbarmak (Kaz, Uzb, Kir, Tgk), Başbarmak (Tuk)	A meat-and-noodle dish consisting of finely chopped boiled meat, noodles, and onion sauce
	Udon	-	Thick wheat noodles prepared and served with soy sauce, scallions, meat, or vegetables
	Doner	Doner/shaurma (Kaz, Uzb), Doner/shaverma (Kir), Doner (Tgk), Donor/şaurma (Tuk)	A dish made of meat, fresh vegetables, and signature sauce, served in lavash
	Dapanji	-	A dish made with a mix of chicken, vegetables, and spices, creating a unique flavor combination. Traditionally, it is served with a side of rice
	Khachapuri	-	A dish made of bread with a cheese filling
	Beef Stroganov	-	A dish made from thinly sliced beef in a savory sauce, served as a side dish with pasta or mashed potatoes
Soups	Borscht	-	A hearty beet-based soup, often served with sour cream
	Manpar	Manpar (Kaz, Uzb), manpar/mampar (Kir, Tgk)	A soup with hand-pulled noodles, meat, and vegetables
	Okroshka	Okroshka (Kaz, Kir, Tgk, Tuk), Chalop/okroshka (Uzb)	A cold soup made with kefir, cucumbers, radishes, eggs, and potatoes
	Tom Yam	-	A spicy and sour soup with shrimp, mushrooms, and fragrant herbs
	Ramen	-	A noodle soup served with broth, meat, eggs, and vegetables
	Shorpa	Shorpa (Kaz), Sho‘rva (Uzb), Shorpo (Kir), Shurbo (Tgk), Chorba (Tuk)	A lamb soup with large-cut vegetables, including tomatoes and bell peppers, seasoned with herbs and spices.
	Kespe	Kespe (Kaz, Kir), Kesma (Uzb), Lagmon/Suyuq osh (Tgk), Kesme (Tuk)	A soup made with egg noodles cooked in a meat broth
	Lentil Soup	Jasymyq sorpasy (Kaz), Yasmiq sho‘rva (Uzb), Jasymyk shorpo (Kir), Shurbo nask (Tgk), Merjimek çorba (Tuk)	A thick, nutritious, and naturally meat-free soup with a mild flavor, typically enhanced with lemon juice and a spiced sauce
	Fish Soup	Balyq sorpasy (Kaz), Baliq sho‘rva (Uzb), Balyk shorpo (Kir), Shurboi mohi (Tgk), Balyk çorbasy (Tuk)	A type of soup where the main ingredient is fish or seafood, complemented by vegetables and grains. It has a rich, vibrant taste, further enhanced by aromatic spices
	Instant Noodles	Jyldam daıyndalatyn kespe (Kaz), Tez tayyor bo‘ladigan lag‘mon (Uzb), Zamatta kesme (Kir), Lapshai favri (Tgk), Tiz taýýar tagam/noodles (Tuk)	Quick-cooking noodles that are prepared in 3–5 min, typically with flavoring powder and seasoning oil
Meat Dishes	Koktal	Koktal (Kaz, Kir, Tuk, Tgk), Ko‘ktal (Uzb)	Smoked fish, typically served with vegetables.
	Naryn	Naryn (Kaz, Kir, Tgk, Tuk), Norin (Uzb)	A dish of thinly sliced boiled meat and hand-pulled noodles
	Shashlyk Beef	Sıyr etinen jasalǵan káýap (Kaz), Mol go‘shtli shashlik (Uzb), Uy etinen jasalğan şaşlık (Kir), Kabobi gushti gov (Tgk), Sygyr etinden şaşlyk (Tuk)	Skewered and grilled chunks of beef, similar to kebabs
	Shashlyk Chicken	Taýyq etinen jasalǵan káýap (Kaz), Tovuq go‘shtli shashlik (Uzb), Took etinen jasalğan şaşlık (Kir), Kabobi murgh (Tgk), Towuk etinden şaşlyk (Tuk)	Succulent chicken meat marinated in spices, skewered, and cooked over an open flame for a smoky, flavorful finish
	Lula Kebab	Lula kebab (Kaz, Kir, Tuk), Lula kabob (Uzb, Tgk)	A dish made from minced meat, shaped onto a skewer, and grilled over charcoal. Served with fresh vegetables and marinated onions
	Shashlyk	Káýap (Kaz), Kabob (Uzb, Tgk), Kebep (Kir), Kebob (Tuk)	General term for skewered and grilled meat
	Beef Cutlet	Siyr etinen kotlet (Kaz), Mol go‘shtli kotlet (Uzb), Uy etinen kotlet (Kir), Kotleti gushti gov (Tgk), Sygyr etinden kotlet (Tuk)	Pan-fried patties made from ground beef and seasonings
	Kazan Kebab	Qazan kebab (Kaz, Kir), Qozon kabob (Uzb), Kabobi qozon (Tgk), Gazan şaşlyk (Tuk)	Pieces of tender roasted meat with a golden crust, seasoned with spices and aromatic herbs, cooked in a kazan (a traditional cauldron). Served with potatoes and fresh onions
	Kuyrdak	Kuyrdak (Kaz, Tgk), Qovurdoq/Kuyrdak (Uzb), Kuurdak (Kir), Gowurdak (Tuk)	A hearty stir-fry made from meat or offal, combined with potatoes and onions
	Orama	Orama (Kaz), Hanum (Uzb), Oromo (Kir)	A steamed rolled pastry dish consisting of thin dough filled with finely chopped meat and vegetables
	Syrne/Sirne	-	A hearty, aromatic dish featuring tender meat stewed in its own juices without water or oil. Toward the end of cooking, vegetables and spices are added to enrich the flavor
	Sausages	Shujyq (Kaz), Kolbasa (Uzb, Kir, Tuk), Kolbasaho/hasib (Tgk)	A meat product made from either cooked minced meat or ground meat mixed with salt and spices, encased in a casing
Salads	Salad with Mushrooms	Sańyraýqulaq qosylǵan salat (Kaz), Qo’ziqorinli salat (Uzb), Qozu karyn salaty (Kir), Khurish bo zanburuġho (Tgk), Kömelek bilen salat (Tuk)	A flavorful salad made with cooked mushrooms, sometimes including sweet peppers, carrots, and onions
	Carrot Salad	Sábiz salaty (Kaz), Sabzi salatasi (Uzb), Sabiz salaty (Kir), Salati sabzi (Tgk), Sӓwizli salat (Tuk)	A spicy and tangy shredded carrot salad, seasoned with garlic and vinegar
	Tongue Salad	Til salaty (Kaz, Kir), Til salatasi (Uzb), Salati zabon (Tgk), Dil salaty (Tuk)	A salad made with thinly sliced cooked beef or lamb tongue, often with vegetables
	Vinegret	Vinegret salaty (Kaz, Kir, Tuk), Vinegret salatasi (Uzb), Salati Vinegret (Tgk)	A beetroot salad with potatoes, pickles, and peas
	Beet salad	Qyzylsha salaty (Kaz), Qizil lavlagi salatasi (Uzb), Kyzylcha salaty (Kir), Salat az lablabu (Tgk), Şugundyr salady (Tuk)	A beetroot salad with finely chopped garlic, dressed with mayonnaise
	Funchose	-	A dish with starch noodles, fresh vegetables, and meat, dressed with soy sauce and spices
	Herring Salad	Seldi salatı (Kaz, Kir), Sel’d salatasi (Uzb), Salati Sel’d (Tgk), Seldi salaty (Tuk)	A layered salad of herring, boiled vegetables, and mayonnaise, also known as “Herring Under a Fur Coat”
	Fried Aubergines	Quyrılğan baklajandar (Kaz), Qovurilgan baklajan (Uzb), Kuurulgan baklajandar (Kir), Bodinjon biryon (Tgk), Gowrulan baklajan (Tuk)	Slices of eggplant pan-fried or deep-fried for a savory treat
	Vegetables	Kökönister (Kaz), Sabzavotlar (Uzb), Jashylchalar (Kir), Sabzavot (Tgk), Gök önümler (Tuk)	Fresh or cooked plants, typically consumed as sides or main dishes
	Broccoli	-	A green cruciferous vegetable, often steamed or stir-fried
	Fresh Salad	Jaña pіsken kökönіsterden jasalğan salat (Kaz), Yangi sabzavotli salat (Uzb), Jañy jashylchalardan jasalgan salat (Kir), Khurish az sabzavoti taru toza (Tgk), Täze gök önümlerden ýasalan salat (Tuk)	A salad made of fresh vegetables, dressed with vegetable oil. Rich in fiber and a great complement to the main course
	Achichuk	Achichuk (Kaz, Kir, Tuk), Achuchuk (Uzb), Shakarob (Tgk)	A salad made with juicy tomatoes, crispy onions, hot peppers, and basil, commonly served with pilaf
	Greek Salad	Grek salatı (Kaz, Kir), Grek salatasi (Uzb), Khurish Yunonī (Tgk), Grek salaty (Tuk)	A light, low-calorie salad made from fresh vegetables, olives, and “Fetaxa” cheese, dressed with olive oil
	Olivier Salad	Olivier salatı (Kaz, Kir), Olivier salatasi (Uzb), Khurish Olivier (Tgk), Oliwier salady (Tuk)	A salad made from diced boiled potatoes, carrots, pickles, meat or boiled sausage, and peas, all dressed in mayonnaise
	Caesar Salad	Sezar salatı (Kaz, Kir), Sezar salatasi (Uzb), Khurish Sezar (Tgk), Sezar salaty (Tuk)	A salad with grilled chicken filet, cherry tomatoes, iceberg lettuce, croutons, and eggs. Its distinctive flavor comes from a rich dressing and finely grated Parmesan cheese
Snacks	Kirieshki Cheese (croutons)	-	Crunchy cheese-flavored croutons or snacks
	Kirieshki Shashlik (croutons)	-	Crunchy shashlik-flavored croutons or snacks
	Popcorn	-	Popped corn kernels, often served salted or sweetened
	Tuc (cracker)	-	Savory crackers with a buttery taste
	French Fries	Kartofel fri (Kaz, Kir), Kartoshka fri/Qovurilgan kartoshka (Uzb), Kartoshka fri/Kartoška qovurdo (Tgk), Kartofel fri/Qovurylan kartoşka (Tuk)	Thinly sliced and deep-fried potatoes, served as a snack or side dish
	Nuggets	-	Bite-sized pieces of breaded and fried chicken
	Cheese Sticks	Irimshik taıaqshalary (Kaz), Pishloq tayoqchalari (Uzb), Sýr tayakchalary (Kir), Chubhoi panir (Tgk), Peýnir taýaklary (Tuk)	Breaded and fried cheese, often mozzarella
	Cereal	Jügeri ülpekteri (Kaz), Makkajo’xori donalari (Uzb), Jügörü ülpekteri (Kir), Ataroqi juvorimakka(Tgk), Mekgejöwen patragy (Tuk)	Breakfast flakes made from toasted corn. They are usually served with milk, yogurt, or juice. To enhance the flavor, berries, chocolate, honey, or nuts may be added
	Chocolate	-	A confectionery product made from cocoa butter and cocoa powder. Depending on the ingredients, chocolate can be dark, milk, white, or bitter
	Eastern Sweets	Shygys tättileri (Kaz), Sharq shirinliklari (Uzb), Chygysh tattuulary (Kir), Shirinikhoyi Sharq (Tgk), Gündogar süýjüleri (Tuk)	A natural and healthy treat made from various roasted nuts and carefully dried fruits with a rich taste
Bakery and Bread	Tandyr Nan	Tandyr nan (Kaz, Kir), Naabay nan (Kir), Tandir non (Uzb), Non-i tandoor (Tgk), Tamdyr çörek/Tamdyr nan (Tuk)	A flatbread baked in a traditional oven
	Hvorost	-	Crispy fried dough pastries, often dusted with powdered sugar
	Borek	Börek (Kaz, Kir, Tgk, Tuk), Boʻrek (Uzb)	A baked or fried pastry filled with meat, cheese, or vegetables
	Ciabatta	-	A white bread with a crisp crust and airy, soft interior, often used for sandwiches
	Bauyrsak	Bauyrsak (Kaz), Boorsok (Kir), Boʻgʻirsoq (Uzb), Orzuq (Tgk), Pişme (Tuk)	Deep-fried dough pieces are shaped into squares or round doughnuts and fried in a cauldron
	Samsa	Samsa (Kaz, Kir, Tuk), Somsa (Uzb), Sambusa (Tgk)	A baked pastry with a savory filling. The crispy texture comes from flaky dough, while the inside contains juicy minced meat, onions, finely chopped vegetables, and spices
	Zheti Kulshe	Shelpek (Kaz), Chalpak (Uzb, Tgk), May tokóç (Kir), Çörek/Çalpak (Tuk)	Airy flatbreads fried in hot oil within seconds
	Cheburek	Shibórek (Kaz), Chiburek (Uzb), Cheburek (Kir, Tgk), Çeburek (Tuk)	Deep-fried pastry filled with minced meat and onions. It is crispy on the outside and juicy inside
	Bukteme	-	Fried pastries filled with liver and potatoes, served with melted butter
	Sandwich	-	A type of open-faced bread dish consisting of slices of bread with meat, cheese, and vegetables placed between them, dressed with spicy sauces
	Toast	-	A porous white bread with a thin crust, toasted on both sides
	Pie	Bälish (Kaz), Pirog (Uzb, Kir, Tgk, Tuk)	A baked or fried dish made of dough with various fillings, including meat, fish, vegetables, or sweet ingredients
Side dishes	Grilled Vegetables	Grildегі kókөníster (Kaz), Qovurilgan sabzavotlar (Uzb), Kuurulgan jashylchalar (Kir), Sabzavoti grillī (Tgk), Panjara gök önümler (Tuk)	Fresh vegetables cooked over a grill for a smoky and caramelized flavor
	Rice	Kúrysh (Kaz), Guruch (Uzb), Kúrych (Kir), Birinj (Tgk), Tüwi (Tuk)	A versatile grain served as a side or main dish, cooked plain or flavored
	Mashed Potato	Kartop ezbesi (Kaz), Kartoshka pyuresi (Uzb), Kartofel pyuresi (Kir), Pure kartosh (Tgk), Kartoşka püresi (Tuk)	Creamy potatoes mashed with butter, milk, or cream
	Buckwheat	Qaraqúmyq (Kaz), Grechka (Uzb, Kir), Marjumak (Tgk), Greçka (Tuk)	A gluten-free grain cooked as a porridge or side dish, rich in nutrients and earthy in flavor
Beverages	Sparkling Water	Gazlangan su (Kaz), Gazlangan suv (Uzb), Gazlangan suu (Kir), Obi gasdor (Tgk), Gazly suw (Tuk)	Carbonated water is often served as a refreshing alternative to still water
	Black Tea	Qara shay (Kaz), Qora choy (Uzb), Kara chai (Kir), Choy siyoh (Tgk), Gara çaý (Tuk)	A strong, fully oxidized tea with a bold flavor, often enjoyed plain or with milk and sugar
	Cappuccino	-	A coffee drink made with equal parts espresso, steamed milk, and milk foam
	Green Tea	Kók shay (Kaz), Koʻk choy (Uzb), Kók chai (Kir), Choy kabud (Tgk), Gök çaý (Tuk)	A lightly oxidized tea with a delicate, earthy flavor, known for its health benefits
	Fanta	-	A fizzy orange-flavored soft drink
	Lemonade	-	A sweet and tangy drink made with lemon juice, water, and sugar
	Water	Su (Kaz), Suv (Uzb), Suu (Kir), Ob (Tgk), Suw (Tuk)	A basic and essential drink for hydration, available as still or sparkling
	Tea with Milk	Süt qosılğan şay (Kaz), Sutli choy (Uzb), Süt qoşulğan chai (Kir), Choy bo shir (Tgk), Sütli çaý (Tuk)	A spiced tea brewed with milk, sugar, and aromatic spices like cardamom and cinnamon
	Shubat	Shubat (Kaz, Kir, Tgk), Qumron (Uzb), Çal (Tuk)	A fermented dairy drink made from camel’s milk
	Belize tea	Belize shay (Kaz), Belize choyi (Uzb), Belize chayi (Kir), Belize choy (Tgk), Belize çäýi (Tuk)	This tea blend uses leaves from Belize’s favorite plants—avocado and cream apple—combined with lime leaves to create a powerful, nutrient-rich tea
	Apple Juice	Alma shyryny (Kaz), Olma sharbati (Uzb), Alma shiresi (Kir), Obi seb (Tgk), Alma şiresi (Tuk)	A fruit juice obtained by pressing apples. Naturally sweet due to its natural sugars, refreshing, and the most common type of juice
	Multifruit Juice	Kóp jemisti shyryn (Kaz), Ko‘p mevali sharbat (Uzb), Köp jemish shiresi (Kir), Sharbati bisersohavi (Tgk), Köp miweli şiresi (Tuk)	A beverage containing juice from several different fruits. It is most commonly made from oranges, bananas, apples, peaches, kiwis, and mangoes
	Coke	-	A carbonated soft drink flavored with vanilla, cinnamon, citrus oils, and other aromas. It has a rich coffee-like taste with caramel notes
	Pomegranate Juice	Anar shyryny (Kaz), Anor sharbati (Uzb), Anar shiresi (Kir), Obi anor (Tgk), Nar şiresi (Tuk)	A fruit juice obtained by pressing the pulp of cultivated pomegranates. It has a bright pink or red color and a refreshing sweet-and-sour or tart taste
	Kymyz	Qymyz (Kaz), Qimiz (Uzb, Tgk), Kymyz (Kir, Tuk)	A traditional drink made from fermented mare’s milk. It has a tangy taste and light fizz and contains natural probiotics and a small amount of alcohol
	Tan	Tan (Kaz, Uzb, Kir, Tgk, Tuk)	A fermented dairy drink made from cow’s or goat’s milk with a starter culture of lactic acid bacteria, dairy yeast, water, and salt
	Sprite	-	A carbonated soft drink with lime and lemon flavor
	Compote	Kompot (Kaz, Uzb, Kir, Tgk, Tuk)	A light, non-alcoholic fruit or berry drink made from dried, frozen, or fresh fruits and berries
	Sorpa	Sorpa (Kaz, Uzb, Kir, Tgk, Tuk)	A liquid dish made with a rich broth by boiling meat on the bone with vegetables, pepper, and spices
	Kefir	Kefir (Kaz, Uzb, Kir), Gatyk (Tuk), Jurghot (Tgk)	A fermented dairy drink made from whole or skimmed cow’s milk, cultured with kefir grains
Desserts	Apple strudel	Alma strýdeli (Kaz, Kir, Tuk), Olma strudel (Uzb), Shtrudeli seb (Tgk)	A pastry filled with spiced apples, raisins, and sometimes nuts, wrapped in thin dough
	Balqaymaq	Balqaimaq (Kaz), Balqaymaq (Uzb, Tgk, Tuk), Balkaymak (Kir)	A dairy product similar to clotted cream, rich and creamy in texture
	Qurt	Qurt (Kaz), Kurut (Kir), Qurut (Uzb, Tgk), Gurt (Tuk)	A dairy product made from fermented milk, with a distinct sour-milk taste and a dense texture
	Irimshik	Irimshik (Kaz), Pishloq (Uzb), Sir (Kir), Panir (Tgk), Peýnir (Tuk)	A national delicacy made from curd, prepared by slow-cooking sour milk for several hours. It has a unique golden-brown color and a sweet, creamy taste
	Cottage cheese	Tvorog (Kaz, Uzb, Kir, Tgk), Chekize (Tuk)	A solid dairy product with a white color, produced by fermenting milk and removing the whey
	Baklava	Baklava (Kaz, Kir, Tgk, Tuk), Paxlava (Uzb)	A Middle Eastern dessert made with layers of filo pastry, nuts, and sweet syrup or honey
	Ice Cream	Balmuzdaq (Kaz), Muzqaymoq (Uzb), Balmuzdak (Kir), Yakhmos (Tgk), Doňdurma (Tuk)	A frozen dessert made from cream, milk, and sugar, available in a variety of flavors
	Chak Chak	Shak-shak (Kaz), Chak-chak (Uzb, Kir, Tgk, Tuk)	A pastry dessert made of deep-fried dough pieces soaked in honey syrup, typically served with tea
	Napoleon	-	A multi-layered puff pastry cake filled with buttercream or custard
	Cheesecake	Cheesecake (Kaz, Uzb, Kir, Tgk), Peýnirli kek (Tuk)	A dessert that varies from a cottage cheese-based baked dish to a soufflé-like treat
	Zhent	Zhent (Kaz, Kir, Tuk), Jent (Uzb, Tgk)	A sweet made from roasted millet, clarified butter, honey, raisins, and nuts
	Syrniki	Syrniki (Kaz, Kir, Tuk), Sirnik (Uzb, Tgk)	Fried curd pancakes, shaped into small patties or rounds, served hot with jam, fruit preserves, condensed milk, or sour cream
	Bliny	Quimaq (Kaz), Qatlama (Uzb), Kuimak (Kir), Quymoq (Tgk), Blin (Tuk)	Thin pancakes, served plain or filled with sweet or savory ingredients

## Data Availability

The original contributions presented in this study are included in the article. Further inquiries can be directed to the corresponding author. The Central Asian Visual Food Atlas is available on GitHub (https://github.com/Central-Asian-Food-Innovation-Lab/Central-Asian-Digital-Visual-Food-Atlas, accessed on 27 July 2025).

## Abbreviations

The following abbreviations are used in this manuscript:

PSEA	Portion size estimation aid
PSEE	Portion size estimation element
NCD	Noncommunicable disease
NHANES	National Health and Nutrition Examination Survey
EFSA EU	European Food Safety Authority European Union
24HR	24-h recalls
FFQ	Food frequency questionnaire
CAFD	Central Asian Food Dataset
CAFSD	Central Asian Food Scenes Dataset
